# Engineering a solid-state metalloprotein hydrogen evolution catalyst

**DOI:** 10.1038/s41598-020-60730-y

**Published:** 2020-02-28

**Authors:** Trevor D. Rapson, HyungKuk Ju, Paul Marshall, Rosangela Devilla, Colin J. Jackson, Sarbjit Giddey, Tara D. Sutherland

**Affiliations:** 1 Health and Biosecurity, CSIRO, Canberra, 2601 ACT Australia; 2 Energy, CSIRO, 3169 Melbourne, VIC Australia; 3 Research School of Chemistry, Australian National University, Canberra, 2601 ACT Australia; 4 Agriculture and Food, CSIRO, Canberra, 2601 ACT Australia

**Keywords:** Electrocatalysis, Bioinspired materials, Fuel cells, Energy, Metalloproteins

## Abstract

Hydrogen has the potential to play an important role in decarbonising our energy systems. Crucial to achieving this is the ability to produce clean sources of hydrogen using renewable energy sources. Currently platinum is commonly used as a hydrogen evolution catalyst, however, the scarcity and expense of platinum is driving the need to develop non-platinum-based catalysts. Here we report a protein-based hydrogen evolution catalyst based on a recombinant silk protein from honeybees and a metal macrocycle, cobalt protoporphyrin (CoPPIX). We enhanced the hydrogen evolution activity three fold compared to the unmodified silk protein by varying the coordinating ligands to the metal centre. Finally, to demonstrate the use of our biological catalyst, we built a proton exchange membrane (PEM) water electrolysis cell using CoPPIX-silk as the hydrogen evolution catalyst that is able to produce hydrogen with a 98% Faradaic efficiency. This represents an exciting advance towards allowing protein-based catalysts to be used in electrolysis cells.

## Introduction

Hydrogen is a valuable commodity currently necessary for fertiliser production, in petroleum refining and in chemical and metallurgical industries. The combustion of hydrogen proceeds with higher efficiency than that of other fuels, and has the added advantage that water is the only reaction product. Consequently, hydrogen is being recognised as an important energy carrier^1^, for power generation in internal combustion engines, gas turbines and fuel cells and to enrich natural gas networks^2^.

Around 60 million tonnes per annum of hydrogen is currently produced primarily by natural gas reforming or coal gasification processes. Carbon dioxide is an unavoidable by-product of these processes^3,4^. Therefore carbon capture and storage would need to be added to the process to make it carbon neutral^2^.

Water electrolysis is the conversion of water to oxygen (O_2_) and hydrogen (H_2_) due to the passage of an electric current. An important advantage of water electrolysis is that the process can be powered by renewable energy, thereby allowing clean hydrogen production. Currently, economic issues limit the portion of global hydrogen production *via* water electrolysis to ~4%^4,5^. There are other alternatives for hydrogen production such as photocatalytic water splitting^6^ and microbial electrolysis cells^7^, however, these are still in the research stages of development.

Water electrolysis requires hydrogen evolution reaction (HER) catalysts to convert protons into H_2_. Currently noble metal catalysts (i.e. platinum group metals) are used as HER catalysts to achieve high hydrogen production rates through the minimization of electrode overpotential to maintain sustained performance and durability^5^. In practice, high catalyst loadings are required in the cells to achieve low cell voltages at high current densities and prolonged lifetime of the cells. There is a worldwide R&D effort to reduce or eliminate the use of platinum in the cells by exploring non-noble metals as catalysts^8,9^. To this end catalysts such as MoS_2_^10,11^ and Ni_5_P_4_^12^ have been investigated and are reported to have activity approaching the activity of platinum based catalysts.

Nature has developed highly efficient biological catalysts to produce hydrogen using first row transition metals such as iron and/or nickel. One such example is hydrogenase enzymes which evolve H_2_ from aqueous solutions with high turnover frequencies (10,000 s^−1^) at ambient temperatures and zero overpotential^13^. Unfortunately, these enzymes do not have sufficient stability outside of their biological context, especially under aerobic conditions, to be useful in industrial applications^14^. In recent years a number of proteins have been designed that mimic the activity of natural hydrogenases and address the instability of hydrogenases in the presence of oxygen. In particular designs where cobalt complexes are incorporated within a protein scaffold (such as cytochromes^15,16^, myoglobin^17^ or synthetic mini-proteins^18–20^) are able to carry out HER under aerobic conditions^19^. Unfortunately many of these engineered proteins require high overpotentials and have poor operational stability^18^.

**Figure 1:**
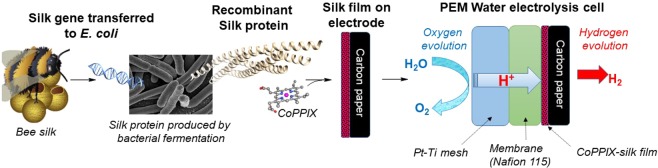
Scheme outlining the preparation of biologically-inspired hydrogen evolution catalysts. CoPPIX – cobalt protoporphyrin IX.

To produce robust catalysts that incorporate the advantages of biological systems and are suitable for industrial applications we have pursued a synthetic biology approach to produce solid-state metalloprotein materials^21,22^. Key to our approach is the use of a recombinant silk protein from honeybees, AmelF3 (Fig. 1). Under appropriate conditions the recombinant silk protein self assembles into an alpha-helical molecular structure with multiple proteins coming together to form a coiled coil, a structure where the alpha helices twist around each other to shield the hydrophobic side of the helices from the aqueous solvent^23^. The structure provides an ideal binding site for metal macrocyles^21^. We have demonstrated that iron protoporphyrin IX (FePPIX, heme *b*) and zinc phthalocyanine tetrasulfonic acid bind to the silk protein^24^. Immobilisation within the silk protein has been found to significantly stabilise the macrocycle by ensuring that the macrocycle is in a monomeric form and preventing overlap of the catalytic sites^25,26^. Furthermore residues within the hydrophobic core of the coiled-coil structure formed by AmelF3 silk provide axial ligation to the metal ion^21,27^.

Importantly recombinant honeybee silk can be produced at a high yield (>10 g L^−1^) using bacterial fermentation and fabricated into stable materials^23,28^. When FePPIX-silk films are cast on glassy carbon electrodes direct electron transfer can be achieved between the electrode and a metal centre incorporated within the silk^26^. Using this approach we have produced an oxygen reduction catalyst and a nitric oxide sensor^29,30^.

Here we report non-noble metal HER electrocatalyst using cobalt protoporphyrin IX (CoPPIX) entrapped within a silk protein. Through protein engineering we modified the axial coordination of the porphyrin to enhance the catalytic properties of the cobalt. We then used the engineered CoPPIX-silk as the non-noble HER catalyst in a water electrolysis cell to demonstrate the robustness of the biologically derived material.

## Results

### Developing a hydrogen producing CoPPIX-silk film

**Figure 2:**
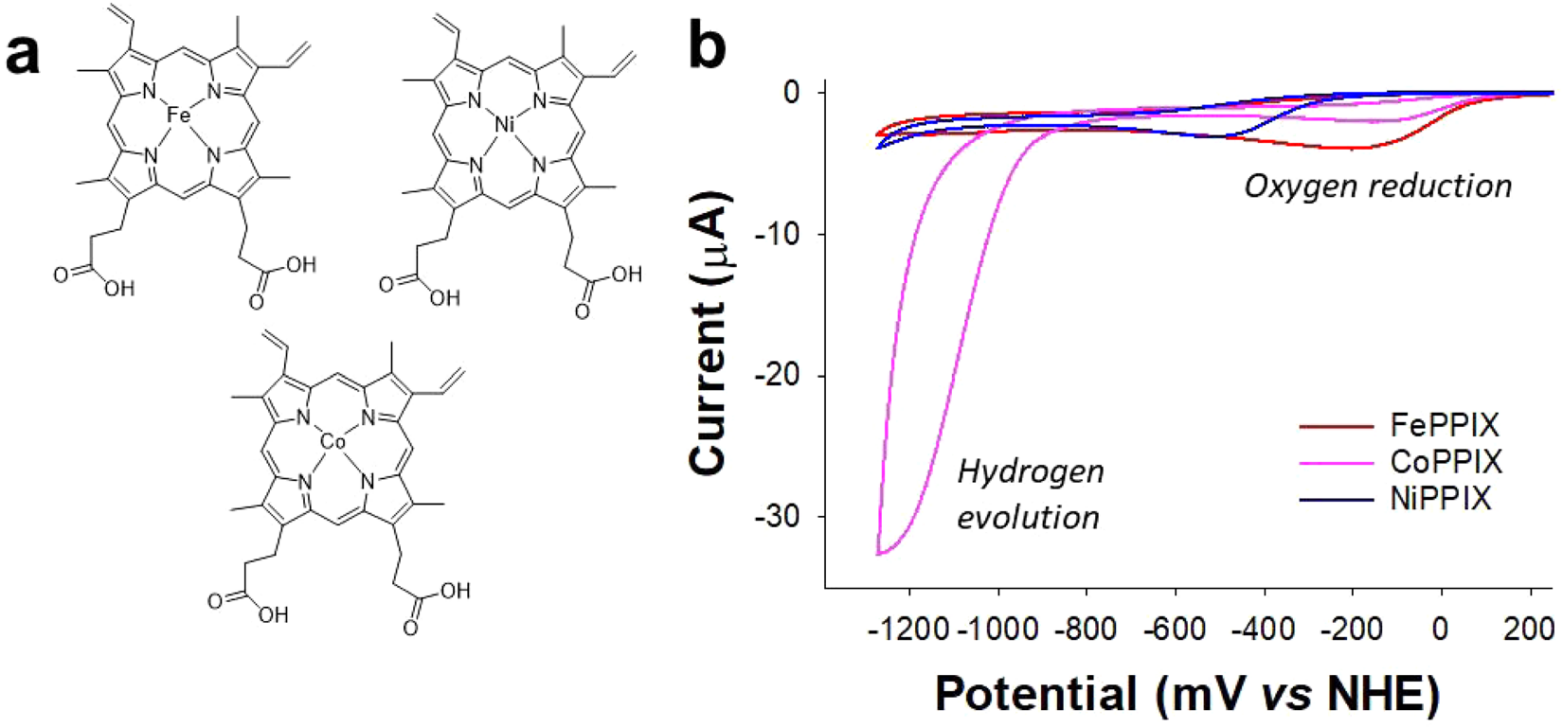
Preparing and testing CoPPIX-silk films for hydrogen evolution. (**a**) The molecular structures of cobalt protoporphryin (CoPPIX), hemin (FePPIX) and nickel protoporphyrin (NiPPIX). (**b**) Cyclic voltammetry of CoPPIX-silk, FePPIX-silk and NiPPIX–silk films on a glassy carbon electrode. Scan rate = 10 mV s^−1^, pH 7.

Initially we sought to determine if hydrogen evolution would be observed when CoPPIX (Fig. 2a) was incorporated into AmelF3 silk films. Cyclic voltammetry (CV) of CoPPIX-silk films on a glassy carbon electrode at pH 7 showed a Faradaic current with an onset potential of −0.74 V and half wave-potential of −1.08 V (Fig. 2b). This was in contrast to that observed when either FePPIX or NiPPIX was incorporated into the silk film (Fig. 2b). The Faradaic current was not observed in control experiments with an unmodified glassy carbon electrode or an electrode modified only with silk and no CoPPIX. (Supplementary Fig. S1). Gas chromatography analysis confirmed that H_2_ gas was produced during bulk electrolysis by CoPPIX-silk films (Supplementary Fig. S2).

Unlike that observed for FePPIX into AmelF3 silk films^26^, no redox couple for Co(III/II) was noted when CoPPIX was incorporated into silk films. This is due to the low spin state of Co(III) and its high reorganisation energy and slow kinetics when reduced to Co(II)^31^. The Co(II/1) couple was not observed as a result of the role of Co(I) in HER^32^. This result is consistent with that reported by Bren and co-workers for cobalt porphyrins in protein scaffolds^18^ and prevents the calculation of turnover frequencies.

### Modifying the silk protein to improve hydrogen evolution

It is known that the axial coordinating ligand plays a strong role in controlling the catalytic properties of the metal centre in haem proteins. The most common coordinating residue in naturally occurring haem protein is histidine (His)^33^. In these proteins, the charge of the His and the strength of the Fe-His bond can be controlled through hydrogen bonding to adjacent amino acid residues^33,34^.

In unmodified AmelF3 silk, a tyrosine residue at position 76 (Tyr76) coordinates to the metal centre of immobilised metal macrocycles. An advantage of using a recombinant protein, rather than relying on the natural sources, is that the protein sequence can be readily manipulated through genetic engineering. We therefore tested the effect of varying the coordinating ligand of the cobalt centre on hydrogen evolution by CoPPIX-silk films.

**Figure 3:**
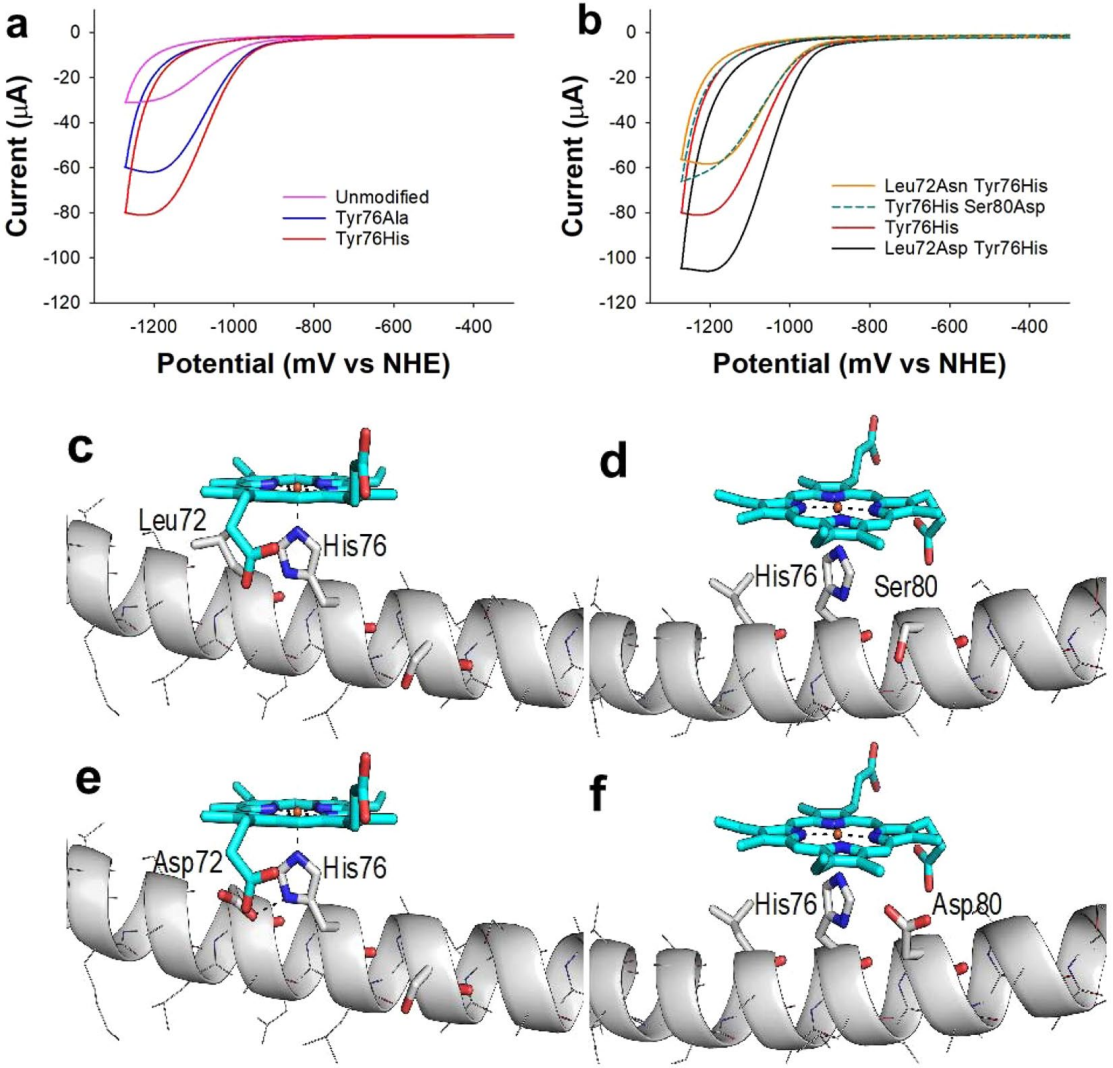
Using protein engineering to enhance hydrogen evolution (**a**,**b**) Cyclic voltammetry of CoPPIX-silk films on a glassy carbon electrode when the axial coordination was altered. Scan rate = 10 mV sec^−1^, pH 7. (**c**–**f**) 3-dimensional model of CoPPIX binding to honeybee silk variants to rationalise results observed in cyclic voltammetry. (**c**,**d**) The Tyr76His variant showing the two rotamers of His76 that allow CoPPIX coordination without steric clashes. (**e**) Leu72Asp Tyr76His variant which could potentially form a hydrogen bond to His76. (**f**) The Tyr76His Ser80Asp variant which cannot adopt conformations that allow hydrogen bonding to His76.

Two variants were tested; firstly, the tyrosine at position 76 was replaced with an alanine (Ala) residue (Tyr76Ala). This substitution would remove coordination by the silk protein leaving a vacant coordination site. It is expected that a water molecule would coordinate to the cobalt centre as the proximal ligand in this case. Secondly, the tyrosine was substituted for a histidine (Tyr76His). Both these substitutions were found to lead to an increase in the Faradaic current from −30 µA to −60 µA for Tyr76Ala and −80 µA for the Try76His variant (Fig. 3a). Tyrosine is an anionic ligand while water and histidine are neutral^35,36^. Our results indicate that the charge of the coordinating ligand plays an important role in the efficiency of hydrogen evolution with neutral residues favoured over negatively charged ligands.

In addition, we altered the residues within hydrogen bonding distance of His76 as these substitutions were predicted to alter the charge of the His and vary the strength of the metal-His coordination bond. Given the alpha-helical molecular structure of honeybee silk, these residues are located exactly four amino acids away in the protein sequence, in either the N or C terminal direction from the histidine (Fig. 3c,d). We therefore tested the effect of the leucine (Leu) and serine (Ser) residues at positions 72 and 80 respectively for the hydrogen bonding residues aspartic acid (Asp) and asparagine (Asn) within the Tyr76His variant.

When an aspartic acid replaced serine at position 80 (Ser80Asp) or when an asparagine (Leu72Asn) was substituted, a decrease in Faradaic current from −80 µA to −60 µA was noted (Fig. 3b). In contrast the Leu72Asp Tyr76His variant showed an increase in Faradaic current to −110 µA. The 3-Dimensional model of Tyr76His AmelF3 silk binding to CoPPIX indicates that an Asp in position 72 could potentially form a hydrogen bond to His76 whilst an Asp residue in position 80 cannot adopt a position to form hydrogen bonds to His76 (Fig. 3e,f). In addition, asparagine is predicted to be a weaker hydrogen bonding partner than aspartic acid which could explain why no enhancement was observed with this substitution (Fig. 3b).

### Demonstration of CoPPIX-silk catalyst in a proton exchange membrane (PEM) water electrolysis cell

The HER catalysts developed here could be used in a range of different hydrogen production processes such as photocatalytic water splitting^6^ or microbial electrolysis cells^7^. To demonstrate the usefulness of our catalyst we chose to test the catalysts in a water electrolysis cell as these systems are already used for commercial hydrogen production. Water electrolysis cells have two electrodes; the anode where the oxygen evolution reaction (OER, Eq. 1) occurs and the cathode where the hydrogen evolution reaction (HER, Eq. 2) takes place. These electrodes are typically separated by a polymer electrolyte membrane, which conducts protons and acts as an insulator^3,5^.

1 $${H}_{2}O\to 2{H}^{+}+\frac{1}{2}{O}_{2}+2{e}^{-}$$

2 $$2{H}^{+}+2{e}^{-}\to {H}_{2}$$

When preparing electrolysis cells, carbon paper is normally used as a support, onto which the catalysts (HER side) are coated. The carbon paper is coated with a hydrophobic polymer such as polytetrafluoroethylene (PTFE) to improve the release of H_2_ from the pores of the carbon paper^37^. In order to produce a water electrolysis cell using a CoPPIX-silk HER catalyst, a procedure for modifying carbon paper electrodes was developed. Hexafluoroisopropanol (HFIP) was used as the solvent to dissolve the silk rather than water to allow efficient wetting of the hydrophobic carbon paper. In addition, carbon black was mixed with the silk, to match the traditionally used platinum on carbon HER catalysts used.

**Figure 4:**
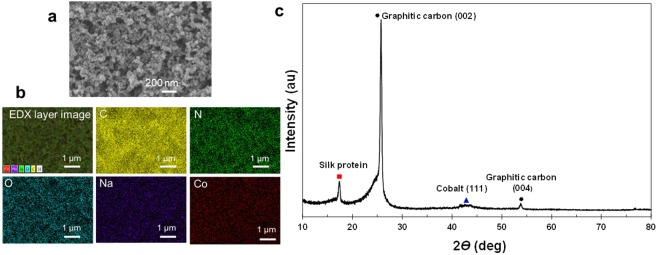
Characterization of optimal carbon paper electrode preparation, CoPPIX-silk film mixed with carbon black (0.5 mg cm^−2^) 5 wt% PTFE coating. (**a**) High resolution FE-SEM image with (**b**) SEM image and corresponding EDX elemental mapping. (**c**) XRD pattern of the CoPPIX-silk catalyst layer.

The silk modified carbon paper electrodes were characterized by high resolution field emission scanning electron microscopy (FE-SEM) imaging and energy dispersive X-ray (EDX) elemental analysis. The silk and carbon black particles appear to be uniformly distributed on the surface of the carbon paper with a particle size around 60–80 nm (Fig. 4a). EDX mapping of the bulk composition confirmed that CoPPIX is evenly dispersed throughout the silk layer (Fig. 4b).

XRD was performed to determine the changes in crystallinity and phases present in the as-prepared electrode (Fig. 4c). The XRD patterns exhibit a strong characteristic peak located at around 26° and another peak located at around 54°, corresponding respectively to the (002) and (004) planes of graphitic carbon. Interestingly, a week broad peak appears around 44°. This is likely due to the protein-cobalt complexes incorporated in the silk. Another sharp peak at around 17° seems to correspond to silk film at (200)^38,39^.

A single PEM electrolysis cell was fabricated with an anode comprised a platinized titanium (Pt-Ti) mesh and cathode comprised of carbon paper coated with the CoPPIX-silk catalyst. The electrodes were separated by a Nafion 115 membrane which acts as the electrolyte providing transport of hydrogen ions from anode to cathode (Fig. 1).

**Figure 5:**
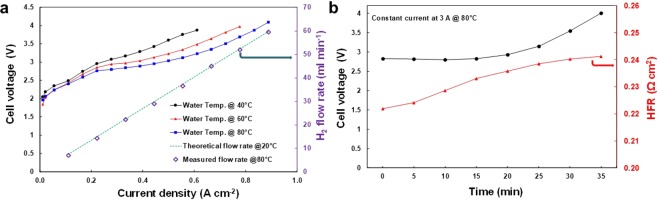
Testing a PEM water electrolysis cell using a CoPPIX-silk as the HER catalyst. (**a**) Polarization curves in a standard water electrolysis cell using CoPPIX-silk based catalyst as a cathode under different temperatures in the range of 40–80 °C. Theoretical and measured hydrogen production rate is plotted (right y-axis) (**b**) Chronopotentiometry loadings of constant current of 3 A at 80 °C of CoPPIX-silk based HER catalyst in the water electrolysis cell.

The cell was operated with deionized tap water at different temperatures (40–80 °C, Fig. 5). At the beginning of the cell evaluation, the pre-conditioning of the cell was carried out by operating the cell at 0.2 A current at 40 °C for 6 h in order to hydrate the polymer membrane. A key parameter to evaluate the efficiency of water electrolysis cells is the Faradaic efficiency of hydrogen production. The hydrogen production rate as expected is linearly proportional to the applied current density, and the measured values as seen in Fig. 5a are well fitted according to Faraday’s law. The Faradaic efficiency at all temperatures and at all points within the polarization curve was close to 98%. The hydrogen production rate was about 60 ml min^−1^ at applied current density of 0.89 A cm^−2^.

A second key parameter is the cell voltage required to reach a current density of 1 A cm^−2^ at 70–80 °C. Typically, PEM electrolysis cells using Pt-based HER catalysts are operated at 1.8–2.2 V to achieve this current density. In Fig. 5a, polarization curves of the electrolysis cell show an onset potential of about 1.85 V. The cell voltage rapidly increases with current density and a voltage of over 4 V would be required to reach a current density of 1 A.cm^−2^. This overpotential behaviour can be attributed to low density of CoPPIX active sites within the silk film, which is an insulating material due to the inherent poor conductivity of proteins^40^.

In order to evaluate the operational stability of the CoPPIX-silk catalyst in the electrolysis cell, a chronopotentiometry test was performed whereby the cell voltage required to drive a constant current of 3 A was monitored for 35 min with the cell held at 80 °C (Fig. 5b**)**. Increases in cell voltage over time indicate degradation of the catalyst. The cell voltage of water electrolysis was stable and remained under 3 V for 20 min after which the voltage was found to begin increasing. In addition to the cell voltage, the ohmic resistance at high frequency of 1 kHz, which mainly corresponded to the polymer membrane resistance of the electrolysis cell, was monitored and found to increase gradually from 0.22 to 0.24 Ω-cm^2^. In contrast to the cell voltage the resistance was noted to increase during the first 20 mins and then stabilised. The increase in cell voltage after 20 mins is most likely a result of deactivation of the Co-PPIX-silk catalyst.

**Figure 6:**
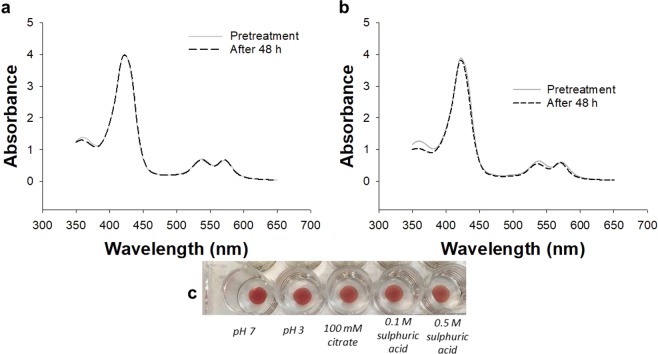
Stability of CoPPIX-silk films in acidic conditions. (**a**,**b**) UV/Vis spectra of CoPPIX-silk before and after the films were immersed in (**a)** 100 mM sodium acetate buffer (pH 3) and (**b**) 0.5 M H_2_SO_4_ for 48 h. (**c**) Photographs of CoPPIX-silk films after immersion in solutions of varying pH.

One possible reason for the deactivation observed after 20 minutes could be the acidic conditions of a PEM electrolysis cell which is within the pH range of 2–4^5^. Many metal macrocycles and proteins are not able to withstand these harsh conditions^41,42^. Whilst we expected the CoPPIX-silk catalyst to be more robust than other systems we tested the films stability by soaking the films in acidic solutions for 48 h. The stability of the films was monitored by visually comparing changes in the films when soaked in different solutions ranging from a pH 7 buffer to 0.5 M sulfuric acid (near pH 0) and through monitoring the UV/Vis spectrum before and after treatment (Fig. 6). No changes were noted in the films after treatment under all conditions tested. This result indicates that the CoPPIX-silk films are stable under strongly acidic conditions and deactivation was not likely due to the pH of the electrolysis cell.

## Discussion

Here we report a HER catalyst based on CoPPIX immobilised within a recombinant silk film (Fig. 1). We modified the silk residue coordinating to the cobalt metal centre and increased the efficiency of the catalyst over three times, indicated by the Faradaic current **(**Fig. 3a,b). Our results indicate that neutral coordinating ligands, such as water or histidine, enhance the rate of the HER. This rate can further be enhanced by strengthening the axial coordination which is possible using hydrogen bonding partners to the axial histidine ligand. Under acidic conditions, such as a PEM electrolysis cell, the coordinating histidine would be protonated and will most likely be replaced by a water ligand. Under these conditions, although a silk residue is not coordinating to the CoPPIX, the silk plays an important role in preventing aggregation of the macrocycles and avoiding overlap of the catalytic sites which is reported to be a contributing factor in the poor stability of metal macrocycle catalysts^25^.

While protein based HER catalysts have been reported by a number of different groups using cobalt macrocycles, the particular advantage of our approach is that we are able to form solid-state protein materials which can be used to fabricate electrode assemblies. The robustness of the silk protein allows organic solvents such as HFIP to be used to allows hydrophobic surfaces such as carbon paper to be modified. In addition, the silk protein is able to tolerate acidic conditions. Here we demonstrate the versatility of the silk-based catalysts by fabricating a PEM water electrolysis cell. While in this work we focused on one specific process for hydrogen production, the HER catalysts here could be employed in a range of different processes and would be especially amenable to applications such as algal electrolysis cells.

When CoPPIX-silk films were tested in a PEM electrolysis cell, high Faradaic efficiencies of 98% were measured. These results are promising first steps in developing a biological HER catalyst. The electrolysis cells fabricated using CoPPIX-silk catalysts showed a high overpotential behaviour, meaning that a higher cell voltage is required to achieve a desirable current density of 1 A cm^−2^. This overpotential is most likely due to the low density of active sites within the CoPPIX-silk films (Supplementary Fig. S3). The reason for this low density of active sites is due to the fact that a single CoPPIX (molar mass 651 g mol^−1^) binds to each silk helix (30,000 g mol^−1^)^21^. One approach to improve the CoPPIX-silk is further protein engineering to design additional binding sites for CoPPIX so that more metal can be incorporated into the AmelF3 silk^27^.

While the CoPPIX-silk based electrolysis cell could be operated for over 30 min, the operational stability would need to be improved if these catalysts are to be used for commercial applications. Our results indicate that the poor operational stability of CoPPIX-silk is not due to the acidic conditions.

Proteins are insulating materials^40^ and it may be possible that the poor conductivity of the silk protein could be the cause of the high ohmic resistance. Recently it has been reported that proteins can have high electronic conductance if a reliable contact to the electrode is established^43^. This result is promising for this work, and provides avenues to explore such as optimising both the ratio of carbon black to silk and decreasing the thickness of the silk films to decrease the resistance of the cell and potentially improve the operational stability.

In conclusion, this work demonstrates the potential of using biomimetic protein catalysts in non-biological applications in harsh environments such as PEM water electrolysis cells. The engineered protein was found to be stable in acidic conditions and produce hydrogen with a high Faradaic efficiency (98%). While the activity of the CoPPIX catalyst is still below other non-platinum reports in the literature such as using Co-Mo-S_x_ chalcogels^44^ or nickel and zinc doped nanorods^45^, further development of both the biological catalyst and the associated materials could lead to higher performance in the future. Improvements in the operational stability are required in addition to lowering the overpotential losses related to the catalyst before these CoPPIX-silk catalysts could be considered as viable for use in commercial electrolysers.

## Methods

### Protein preparation and site directed mutagenesis

Recombinant honeybee silk protein, AmelF3, was produced by fermentation in *E*. *coli* as described by Weisman *et al*.^46^. Site directed mutagenesis was carried out using the Q5 Site-Directed Mutagenesis Kit (New England BioLabs) following the manufacturer’s instructions and the sequences of the modified constructs were confirmed by DNA sequencing.

### Glassy carbon electrode preparation

Glassy carbon electrodes (GCEs, 3 mm eDAQ, Sydney) were polished as per the manufacturer’s instructions using 0.05 µm alumina polish followed by sonication for 1 min in milliQ water and 1 min in ethanol. CoPPIX and other metal macrocycle solutions (1 mg mL^−1^) were prepared by dissolving the macrocycle (10 mg) in 1 mL of 0.1 M NaOH and water (9 mL). The macrocycle solution was added to an AmelF3 solution (5 mg mL^−1^) to give a 1:1 molar ratio of metal macrocycle to protein. This solution (10 µL) was cast onto the GCE and left to dry for at least 1 h. The resultant film was soaked in 70% v/v methanol:water for 30 min and left to dry for at least 1 h prior to use.

### Cyclic voltammetry

Cyclic voltammetry was carried out using a BASi Epsilon potentiostat with a C3 cell stand. A three-electrode system was employed comprising a modified glassy carbon electrode as the working electrode, Pt wire counter, and Ag/AgCl-saturated NaCl reference electrode. The standard buffer solution was a 0.1 M sodium phosphate buffer at pH 7.0. All potentials are quoted versus the normal hydrogen electrode (NHE), calculated as Ag/AgCl +196 mV. The general scan procedure was as follows; scan rate: 10 mV s^−1^, initial potential: −600 mV s^−1^, switching point 1: 400 mV, switching point 2: −1500 mV, quiet time: 10 s, total number of scans: 5. The final cycle is reported.

### Bulk electrolysis

A carbon cloth electrode (AvCarb HCB 1071, Ballard Material Products, USA, 22.5 cm^2^) was covered with 1 mL of a 1:1 molar ratio silk:CoPPIX solution (5 mg mL^−1^) prepared as described earlier and dried overnight. The impregnated cloth was soaked in 70% methanol:water for 30 min and left to dry for at least 1 h prior to use. Controlled Potential Electrolysis was carried out using a three-compartment cell in which the counter electrode (Pt wire) was separated from the working electrode by a glass frit. The working electrode (carbon cloth with CoPPIX-silk) was placed in a 0.1 M sodium phosphate buffer at pH 7.0. A fixed potential of −1500 mV vs Ag/AgCl was applied. Prior to electrolysis, the buffer solution was purged with argon gas for 20 min. 1 mL samples of the headspace gas were collected and placed into an exetainer flushed with argon. Gas samples were analysed by a Shimadzu 2014 Gas Chromatograph coupled to a Thermal Conductivity Detector. Peak detection was achieved on a Molsieve 5 A 80/100 column isothermal at 40 °C using argon as carrier gas and detector set at 70 mA. Peak identity was confirmed by retention time against hydrogen standards. Quality control was checked by running blanks and standards for each batch of samples.

### Carbon paper electrode preparation

Carbon paper electrodes (SGL 39BC, thickness: 340 μm, Sigracet^®^) were modified with silk. Given the hydrophobic nature of the surface of the PTFE, freeze dried sponges of AmelF3 were dissolved in hexafluoro isopropanol (HFIP) rather than the protein being prepared in water^47^. Given that CoPPIX is not soluble in HFIP, the macrocycle was incorporated into a precast silk film by soaking the film in a 70:30 v/v methanol:water solution with CoPPIX (1 mg mL^−1^). The effect of using carbon black (Vulcan XC 72, Fuel Cell Store, USA; 0.5 mg cm^−2^) to increase the surface area was also tested. The optimum procedure was found using 5 wt% PTFE with carbon black in HFIP (10 mg mL^−1^) dispersed by sonication for 45 min. The carbon black solution could either be cast as a film prior to the silk or mixed with a solution of silk dissolved in HFIP.

### Water electrolysis cell setup

A water electrolysis cell with a 9 cm^2^ active area was fabricated as reported in previous publications^48^. Briefly, the membrane electrode assembly (MEA) consisted of platinized titanium (Pt-Ti) mesh (thickness: 250 μm, Fuel Cell Store, USA) as an anode (oxygen electrode) and CoPPIX-silk films with 5 wt% PTFE mixed with 0.5 mg cm^−2^ carbon black as a catalyst on carbon paper as the cathode (hydrogen electrode). The MEA was fabricated by mechanically pressing together the prepared electrodes on both sides of a Nafion 115 membrane at a torque of 4 Nm. A Nafion solution (5 wt%, 1100EW, Ion Power, Inc., USA) was sprayed on each electrode surface as an outer ionomer before cell assembly. Experiments of water electrolysis cell were carried out at cell temperatures from 40 °C to 60 °C and under ambient pressure. Water supply to the anode chamber of the cell was deionised tap water (<1 μS cm^−1^) circulated in the oxygen electrode side of the cell. Produced hydrogen gas was passed through a moisture trap and the hydrogen flow rate was carefully monitored by a digital volumetric flow meter (Defender 510, BIOS International Corp., USA). The cell performance was obtained by using a DC power supply (Powerbox PBX, Australia) and a digital multimeter (Keithley 192 DMM). Ohmic part measurement of the electrolysis cell resistance was conducted at real time at a high frequency of 1 kHz using an AC-impedance milliohmeter (3562 HiTester, Hioki, Japan).

### MEA characterization

Surface morphology and elemental qualitative analyses of the electrode catalyst layer of CoPPIX-silk film were performed by field emission scanning electron microscopy (FE-SEM; Merlin-Zeiss Ultra-Plus with a Gemini II column, Germany) equipped with an energy dispersive X-ray spectroscopy (EDX; X-Max 80 mm^2^, Oxford Instruments, UK). The crystallinity and phase analyses of the electrode were performed using X-ray diffraction (XRD; D2 Phaser, Bruker AXS, Germany) with a Cu Kɑ radiation source. Diffraction peak identification was performed with the Eva (Bruker Inc., USA) software using the ICDD crystal database (PDF4+).

## Supplementary information

Supplementary Information.

## References

[1] Staffell I (2019). The role of hydrogen and fuel cells in the global energy system. Energy Environ. Sci..

[2] Voldsund M, Jordal K, Anantharaman R (2016). Hydrogen production with CO2 capture. Int. J. Hydrogen Energy.

[3] Ju HK, Badwal S, Giddey S (2018). A comprehensive review of carbon and hydrocarbon assisted water electrolysis for hydrogen production. Appl. Energy.

[4] Badwal SPS, Giddey S, Munnings C (2018). Emerging technologies, markets and commercialization of solid-electrolytic hydrogen production. Wiley Interdiscip. Rev. Energy Environ..

[5] Carmo M, Fritz DL, Mergel J, Stolten D (2013). A comprehensive review on PEM water electrolysis. Int. J. Hydrogen Energy.

[6] Tentu RD, Basu S (2017). Photocatalytic water splitting for hydrogen production. Curr. Opin. Electrochem..

[7] McCormick AJ (2013). Hydrogen production through oxygenic photosynthesis using the cyanobacterium Synechocystis sp. PCC 6803 in a bio-photoelectrolysis cell (BPE) system. Energy Environ. Sci..

[8] Wang, J., Xu, F., Jin, H., Chen, Y. & Wang, Y. Non-Noble Metal-based Carbon Composites in Hydrogen Evolution Reaction: Fundamentals to Applications. *Adv*. *Mater*. **29** (2017). 10.1002/adma.201605838 28234409

[9] Ledendecker Marc, Mondschein Jared S., Kasian Olga, Geiger Simon, Göhl Daniel, Schalenbach Max, Zeradjanin Aleksandar, Cherevko Serhiy, Schaak Raymond E., Mayrhofer Karl (2017). Stability and Activity of Non-Noble-Metal-Based Catalysts Toward the Hydrogen Evolution Reaction. Angewandte Chemie International Edition.

[10] Zang Y (2019). Tuning orbital orientation endows molybdenum disulfide with exceptional alkaline hydrogen evolution capability. Nat. Commun..

[11] Hu J (2017). Engineering stepped edge surface structures of MoS2 sheet stacks to accelerate the hydrogen evolution reaction. Energy Environ. Sci..

[12] Laursen AB (2015). Nanocrystalline Ni5P4: A hydrogen evolution electrocatalyst of exceptional efficiency in both alkaline and acidic media. Energy Environ. Sci..

[13] Lubitz W, Ogata H, Rüdiger O, Reijerse E (2014). Hydrogenases. Chem. Rev..

[14] Karunadasa HI, Chang CJ, Long JR (2010). A molecular molybdenum-oxo catalyst for generating hydrogen from water. Nature.

[15] Sommer DJ (2016). Reengineering cyt b562 for hydrogen production: A facile route to artificial hydrogenases. Biochim. Biophys. Acta - Bioenerg..

[16] Kandemir B, Chakraborty S, Guo Y, Bren KL (2016). Semisynthetic and Biomolecular Hydrogen Evolution Catalysts. Inorg. Chem..

[17] Bacchi M (2014). Cobaloxime-based artificial hydrogenases. Inorg. Chem..

[18] Firpo V, Le JM, Pavone V, Lombardi A, Bren KL (2018). Hydrogen evolution from water catalyzed by cobalt-mimochrome VI*a, a synthetic mini-protein†. Chem. Sci..

[19] Kleingardner JG, Kandemir B, Bren KL (2014). Hydrogen evolution from neutral water under aerobic conditions catalyzed by cobalt microperoxidase-11. J. Am. Chem. Soc..

[20] Kandemir B, Kubie L, Guo Y, Sheldon B, Bren KL (2016). Hydrogen Evolution from Water under Aerobic Conditions Catalyzed by a Cobalt ATCUN Metallopeptide. Inorg. Chem..

[21] Rapson TD (2015). De Novo Engineering of Solid-State Metalloproteins Using Recombinant Coiled-Coil Silk. ACS Biomater. Sci. Eng..

[22] Rapson T (2016). Solid-State Metalloproteins—An Alternative to Immobilisation. Molecules.

[23] Sutherland TD, Huson MGMG, Rapson TDTD (2018). Rational design of new materials using recombinant structural proteins: Current state and future challenges. J. Struct. Biol..

[24] Horgan C (2016). Phosphorescent oxygen-sensing and singlet oxygen production by a biosynthetic silk. RSC Adv..

[25] Li W, Yu A, Higgins DC, Llanos BG, Chen Z (2010). Biologically inspired highly durable iron phthalocyanine catalysts for oxygen reduction reaction in polymer electrolyte membrane fuel cells. J. Am. Chem. Soc..

[26] Rapson TD, Christley-Balcomb AM, Jackson CJ, Sutherland TD (2020). Enhancement of metallomacrocycle-based oxygen reduction catalysis through immobilization in a tunable silk-protein scaffold. J. Inorg. Biochem..

[27] Rapson TD (2017). Design of silk proteins with increased heme binding capacity and fabrication of silk-heme materials. J. Inorg. Biochem..

[28] Sutherland, T. D., Rapson, T. D., Huson, M. G. M. G. & Church, J. S. Recombinant Structural Proteins and Their Use in Future Materials. in *Fibrous Proteins: Structures and Mechanisms* (eds. Parry, D. A. D. & Squire, J. M.) **82**, 491–526 (Springer, 2017). 10.1007/978-3-319-49674-0_15 28101871

[29] Rapson TD (2017). Bioinspired electrocatalysts for oxygen reduction using recombinant silk films. J. Mater. Chem. A.

[30] Musameh MM, Dunn CJ, Uddin MH, Sutherland TD, Rapson TD (2018). Silk provides a new avenue for third generation biosensors: Sensitive, selective and stable electrochemical detection of nitric oxide. Biosens. Bioelectron..

[31] Marcus R, Sutin N (1985). Electron transfers in chemistry and biology. Biochim. Biophys. Acta.

[32] Artero, V., Chavarot-Kerlidou, M. & Fontecave, M. Splitting water with cobalt. Angew. *Chemie - Int. Ed*. **50**, 7238–7266 (2011). 10.1002/anie.201007987 21748828

[33] Poulos TL (2014). Heme enzyme structure and function. Chem. Rev..

[34] Poulos TL (1996). The role of the proximal ligand in heme enzymes. J. Biol. Inorg. Chem..

[35] Samanta S, Das PK, Chatterjee S, Dey A (2015). Effect of axial ligands on electronic structure and oxygen reduction by iron porphyrin complexes. J. Porphyr. Phthalocyanines.

[36] Amanullah S, Singha A, Dey A (2019). Tailor made iron porphyrins for investigating axial ligand and distal environment contributions to electronic structure and reactivity. Coord. Chem. Rev..

[37] Ju HK, Giddey S, Badwal SPS, Mulder RJ (2016). Electro-catalytic conversion of ethanol in solid electrolyte cells for distributed hydrogen generation. Electrochim. Acta.

[38] Wang QF (2016). Two-dimensional molybdenum disulfide and tungsten disulfide interleaved nanowalls constructed on silk cocoon-derived N-doped carbon fibers for hydrogen evolution reaction. Int. J. Hydrogen Energy.

[39] Wang HY, Zhang YQ (2013). Effect of regeneration of liquid silk fibroin on its structure and characterization. Soft Matter.

[40] Rosenberg B (1962). Electrical conductivity of proteins. Nature.

[41] Yamazaki S (2018). Metalloporphyrins and related metallomacrocycles as electrocatalysts for use in polymer electrolyte fuel cells and water electrolyzers. Coord. Chem. Rev..

[42] Chapman R, Stenzel MH (2019). All Wrapped up: Stabilization of Enzymes within Single Enzyme Nanoparticles. J. Am. Chem. Soc..

[43] Zhang B (2019). Role of contacts in long-range protein conductance. Proc. Natl. Acad. Sci. USA.

[44] Staszak-Jirkovský J (2016). Design of active and stable Co-Mo-Sx chalcogels as pH-universal catalysts for the hydrogen evolution reaction. Nat. Mater..

[45] Ling T (2019). Well-Dispersed Nickel- and Zinc-Tailored Electronic Structure of a Transition Metal Oxide for Highly Active Alkaline Hydrogen Evolution Reaction. Adv. Mater..

[46] Weisman S (2010). Honeybee silk: recombinant protein production, assembly and fiber spinning. Biomaterials.

[47] Rapson Trevor D., Church Jeffrey S., Trueman Holly E., Dacres Helen, Sutherland Tara D., Trowell Stephen C. (2014). Micromolar biosensing of nitric oxide using myoglobin immobilized in a synthetic silk film. Biosensors and Bioelectronics.

[48] Ju HK, Giddey S, Badwal SPS (2017). The role of nanosized SnO2 in Pt-based electrocatalysts for hydrogen production in methanol assisted water electrolysis. Electrochim. Acta.

