# Supplementary material for: Engineering a solid-state metalloprotein hydrogen evolution catalyst
Source: Sci Rep. 2020 Feb 28;10:3774. doi: 10.1038/s41598-020-60730-y (PMC7048781; doi:10.1038/s41598-020-60730-y)
Supplement: Supplementary file 1 — Supplementary Information. [file 41598_2020_60730_MOESM1_ESM.docx]

## Supplementary Information to:

## Engineering a solid-state metalloprotein hydrogen evolution catalyst

Trevor D. Rapson^1*ǂ^, HyungKuk Ju^2ǂ^, Paul Marshall^3^, Rosangela Devilla^4^, Colin J. Jackson^3^, Sarbjit Giddey^2^, Tara D. Sutherland^1^

^1^ CSIRO Health and Biosecurity, Black Mountain, ACT, 2601, Australia

^2^ CSIRO Energy, Private Bag 10, Clayton South, 3169, Victoria, Australia

^3^ Australian National University, Research School of Chemistry, Acton, 2601, ACT, Australia

^4^ CSIRO Agriculture and Food, Black Mountain, ACT, 2601, Australia

*Corresponding author email: [trevor.rapson@csiro.au](mailto:trevor.rapson@csiro.au)

^ǂ^ Authors contributed equally to this work


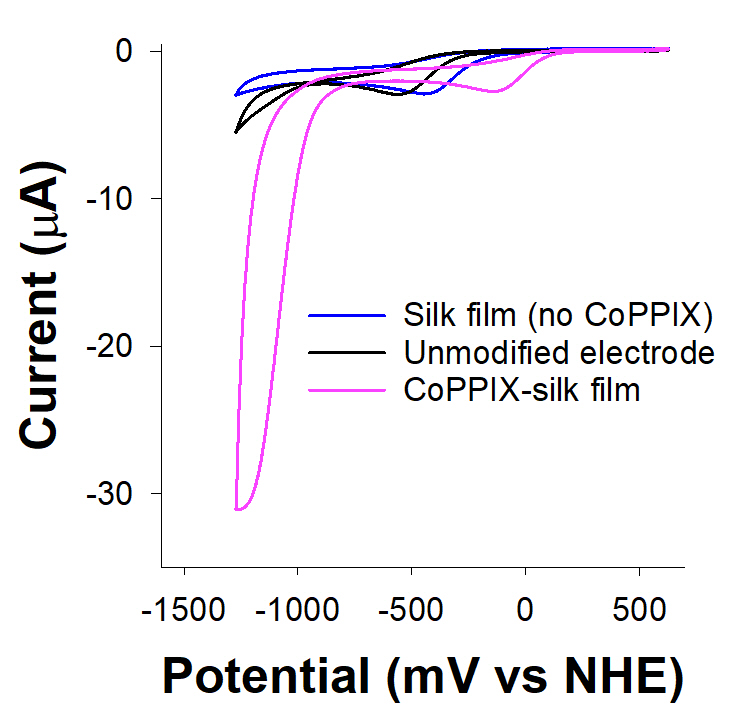


**Fig. S1**. Cyclic voltammograms of control electrodes in comparison to a CoPPIX-silk film. 10 mV sec^-1^, pH 7.


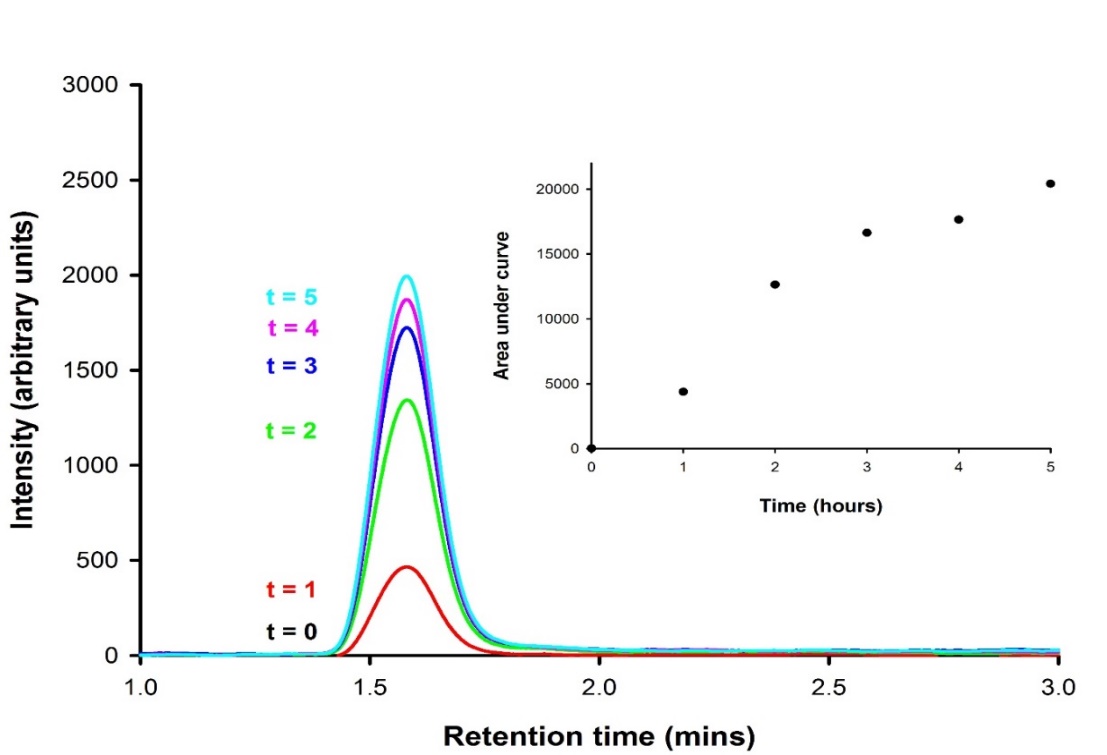


**Fig S2.** Gas chromatography analysis of the headspace from bulk electrolysis of a CoPPIX-silk film cast on carbon cloth (-1.3 V).

**
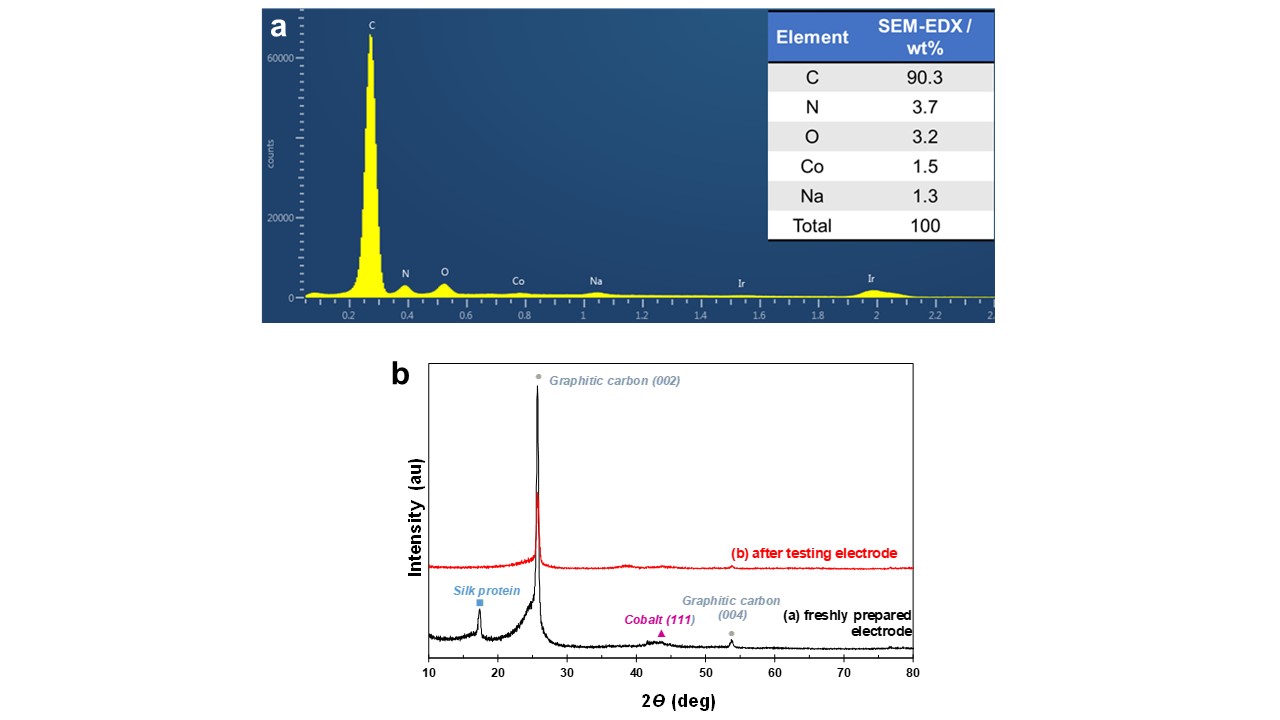
**

**Fig. S3**. Characterization of optimal carbon paper electrode preparation (CoPPIX-silk film mixed with carbon black (0.5 mg cm^-2^) 5 wt% PTFE coating using EDX element analysis.
